# Prediction of telomere length and telomere attrition using a genetic risk score: The multi-ethnic study of atherosclerosis (MESA)

**DOI:** 10.3389/fragi.2022.1021051

**Published:** 2022-10-11

**Authors:** Cecilia Castro-Diehl, Jennifer A. Smith, Wei Zhao, Xu Wang, Bhramar Mukherjee, Teresa Seeman, Belinda L. Needham

**Affiliations:** ^1^ Section of Preventive Medicine and Epidemiology, Department of Medicine, Boston University School of Medicine, Boston, MA, United States; ^2^ Department of Epidemiology and Center for Social Epidemiology and Population Health, School of Public Health, University of Michigan, Ann Arbor, MI, United States; ^3^ Survey Research Center, Institute for Social Research, University of Michigan, Ann Arbor, MI, United States; ^4^ Department of Biostatistics, University of Washington, Seattle, WA, United States; ^5^ Department of Biostatistics, School of Public Health, University of Michigan, Ann Arbor, MI, United States; ^6^ Department of Medicine, Division of Geriatrics, UCLA, Los Angeles, CA, United States

**Keywords:** genetic risk score, telomere length, telomere attrition, race/ethnicity, PCR

## Abstract

**Background:** Short telomere length (TL) and telomere attrition (TA) have been associated with age-related diseases.

**Objective:** We assessed whether a genetic risk score for short TL (GRS-TL) combining seven TL-associated genetic variants identified in a European-ancestry genome-wide association study (GWAS) was associated with TL and TA over 10 years.

**Methods:** Relative TL (T/S ratio) was measured by the quantitative polymerase chain reaction method for a sample of white, African American, and Hispanic participants, who attended Exam 1 and/or 5 of the Multi-Ethnic Study of Atherosclerosis (MESA). Our final sample included 1,227 participants for the TL analysis and 1,138 for the TA analysis. Participants were 45–84 years at Exam 1. We used a linear mixed effects model and adjusted for age, sex, and population structure. Models were stratified by race/ethnicity.

**Results:** In the TL analysis, higher GRS-TL significantly predicted shorter TL (estimates = -0.18 [S.E. = 0.08], *p* = 0.02 for white; -0.18 [0.07], *p* < 0.01 for African American; and -0.13 [0.05], *p* = 0.02 for Hispanic) in fully adjusted models. In the TA analysis, no association between GRS-TL and TA over 10 years was found.

**Conclusion:** Although GRS-TL was developed in European-ancestry populations, it was significantly associated with TL (but not TA) in all three race/ethnic groups examined.

## Introduction

Telomeric DNA shortens with each division of the cell (Fuster and Andres, 2006; Martinez and Blasco, 2011), and telomere shortening occurs throughout life. (Frenck et al., 1998). Telomere length (TL) has been proposed as a biomarker of aging-related diseases. (De Meyer et al., 2018). *In vitro*, telomere shortening correlates with cellular senescence and apoptosis. (Rudolph et al., 1999). In epidemiological studies, the attrition of telomeres has been linked to cardiovascular disease (CVD). Studies have found that shorter telomere length is associated with increased odds of mortality, (Cawthon et al., 2003; Epel et al., 2008; Kimura et al., 2008; Weischer et al., 2012), psychological stress, (Epel et al., 2004), and adverse socioeconomic position. (Needham et al., 2013; Geronimus et al., 2015).

TL shows important variability among individuals. (Frenck et al., 1998; Okuda et al., 2002). At birth, TL varies from 5,000 to 15,000 base pairs, (Sanders and Newman, 2013), with a fast rate of telomere attrition (TA) during growth and development, but by the age of 20, the rate of TA declines gradually. (Aubert and Lansdorp, 2008; Hjelmborg et al., 2015). Men and older people tend to have shorter TL than women and younger people. (Muezzinler et al., 2013). These individual differences appear to result from both environmental and genetic factors. (Okuda et al., 2002). A meta-analysis in over 19,000 subjects reported an estimate of TL heritability of 0.70, (Broer et al., 2013), and a genome-wide association study (GWAS) meta-analysis of more than 37,000 individuals (from 15 cohorts in Europe and Australia, all of European descent) identified seven loci associated with mean TL. (Codd, Nelson et al., 2013).

Although other TL GRSs have been estimated using TL measured by DNA sequencing, our GRS, is the only one that examined TL measured by quantitative polymerase chain reaction method (qPCR) which is the same phenotype as in our study. To our knowledge, only one previous study has examined a genetic risk score for short TL (GRS-TL) as a predictor of TL in a multi-ethnic sample that included whites, African Americans, and Hispanics. (Hamad et al., 2016). However, there is still scant literature about longitudinal measurements of TL. The purpose of the current study was to assess the association of the GRS-TL with both TL and TA over 10 years in the Multi-Ethnic Study of Atherosclerosis (MESA).

## Materials and methods

### Study population

Our sample was derived from the Multi-Ethnic Study of Atherosclerosis (MESA), (Bild et al., 2002), a prospective cohort study of the natural history of CVD. Between 2000 and 2002, MESA recruited 6,814 white, African American, Chinese American, and Hispanic participants aged 45–84 in the United States. Our analytical sample for the TL analysis included 1,162 participants with TL measurement at Exam 1, 1,203 participants with TL measurement at Exam 5, and 1,227 participants with TL measurement at Exams 1 and/or 5 (pooled sample). The analytic sample for the TA analysis included 1,138 participants who had TL measurement at both Exams 1 and 5.

### Telomere length

Stored blood samples from MESA Exams 1 and 5 were used to assess TL. Extensive details of the protocol for blood collection and DNA extraction are described elsewhere. (Carroll et al., 2019). DNA was extracted and purified using sodium dodecyl sulfate cell lysis and salt precipitation methods. DNA was of high quality and high molecular weight. TL assays were performed at the Blackburn Laboratory at the University of California, San Francisco, using the quantitative polymerase chain reaction (qPCR) method to measure TL relative to standard reference DNA (T/S ratio). (Cawthon, 2002; Lin et al., 2010). Each sample was assayed 3 times on 3 different days. (Carroll et al., 2019; Needham et al., 2019). Briefly, sample plates were assayed in groups of three 96 well plates. Any assay run with 8 or more invalid control wells was considered a failed run. The mean of the T/S values was calculated, and values that deviated most from the mean were marked as a potential outlier. The mean of the T/S value was calculated without the potential outlier (99.8% of all samples was free of outliers). DNA samples were coded, and the lab personnel was blind to all other measurements of study participants. The interassay coefficient of variation was 2.9% ± 2.1%. (Carroll et al., 2019).

### Genetic risk score for short telomere length

To create our GRS-TL, we used the effect estimates (betas) reported in the GWAS meta-analysis (Codd et al., 2013) that identified seven loci associated with mean TL including *ACYP2* (*rs11125529*), *TERC* (*rs10936599*), *NAF1* (*rs7675998*), *TERT* (*rs2736100*), *OBFC1* (*rs9420907*), *ZNF208* (*rs8105767*) and *RTEL1* (*rs755017*). These 7 single nucleotide polymorphisms (SNPs) showed a consistent association with short TL at the genome-wide significance (P < 5 × 10^−8^) among individuals of European ancestry. Each SNP explained a very small proportion of the total variance in TL (0.08–0.36%), but the variance in TL explained by the seven SNPs jointly was greater. (Codd et al., 2013). MESA measured genotype data was available from genetic data collected between 2000 and 2013. For SNPs not directly genotyped in MESA (rs10936599, rs7675998, and rs755017), we used imputed genotype by obtaining SNP dosages from 1000 Genomes Project imputed data. The imputation quality was excellent (INFO score >0.92) for all imputed SNPs.

We calculated the GRS-TL by summing the number of risk alleles (from 0 to 2) in the seven loci associated with mean TL and weighted by their effect size in the GWAS meta-analysis. (Codd et al., 2013). A higher GRS-TL indicates a greater genetic risk for short TL.

We created histograms and violin plots to show the distribution of the GRS-TL in the whole sample and in different race/ethnic groups. For violin plots, TL was dichotomized at the 25th percentile, and participants in the bottom quartile (shortest TL) were compared to those in the top three quartiles (longest TL). We plotted the distribution of the GRS-TL in the bottom TL quartile vs. the other quartiles within each race/ethnic group.

### Covariates

Our models were adjusted for study exam (Exam 1 vs. 5) in the TL analysis and for time since Exam 1 in the TA analysis. We also accounted for age in years (at Exam 1 or Exam 5 for the TL analysis and age at Exam 1 for the TA analysis), sex, and genetic principal components (PCs) calculated by MESA that were extracted from genome-wide data. Adding PCs as covariates in regression models accounts for population stratification that can cause type I error inflation and/or loss of power in genetic association studies. (Zhang et al., 2003). We used the PCs calculated within each ethnic group separately for primary analysis and those in the total MESA sample (all race/ethnicities combined) for secondary analysis. The first 3 PCs explained about 78% of the observed variation in the whole sample.

### Statistical analyses

We conducted descriptive analyses to examine the average TL at Exam 1, Exam 5 and change over the 10-year follow-up from Exam 1 to Exam 5 for the full sample and by demographics.

To examine the associations of GRS-TL with TL and 10-year TA in our multi-ethnic sample, our primary analyses were race/ethnicity stratified, and secondarily we performed analyses with all race/ethnicities combined for both the TL and TA analyses.

#### TL analysis

We used a linear mixed-effects model (LMEM) that included all valid observations from Exams 1 and 5 to examine the association between the GRS-TL and TL comparing whites, African Americans, and Hispanics. The model included TL as the outcome and GRS-TL as the main exposure. GRS-TL and covariates were centered at their population means.

Models were adjusted for an exam indicator (reference: Exam 1), age at Exam 1 or 5, sex (reference: female), and PCs specific for each race/ethnic group. An individual-level random intercept was included in the model to account for the within-person correlation of repeated telomere measures (for those who have telomere measures at both exams). Robust standard errors were reported.

In a secondary analysis, we combined all race/ethnic groups and additionally included in the model race/ethnicity (dummy variables for African American and Hispanic with white as the reference category), the interactions between GRS-TL and race/ethnicity, and PCs combined for all race/ethnic groups.

#### TA analysis

We used LMEM to examine the association between the GRS-TL and 10-year TA. Follow-up time since Exam 1 variable was centered on the individual’s average follow-up time to obtain the estimate of the intra-individual change in TL over time. All other covariates were centered on the population mean. The model included follow-up time since Exam 1 and the interaction between follow-up time and the GRS-TL. The coefficient for the interaction term of follow-up time and GRS-TL represents the effect of the GRS-TL on the annual change in TL. The model also included interactions of time with age at Exam 1, sex, and PCs. An individual-level random intercept was included to account for the intra-individual variability of the baseline TL and the change in TL over time. Robust standard errors were reported. For the TA analysis, our primary analysis was also race/ethnicity stratified. In a secondary analysis, we combined all race/ethnic groups. We included three-way interaction terms of the follow-up time, GRS-TL, and race/ethnicity dummy variables. To account for population stratification, we included the first three race/ethnicity-specific PCs for the primary race-stratified analysis and the first three PCs for the combined sample for the secondary combined-race analysis.

## Results

The sample included white (*n* = 323, 28%), African American (n = 330, 28%), and Hispanic participants (n = 509, 44%) at Exam 1 (Table 1) with an average age of 61 years at Exam 1 and 70 years at Exam 5.

**TABLE 1:** Characteristics of study participants.

	Exam 1 TL sample	Exam 5 TL sample	10-Year TA sample	
			Exam 1	Exam 5
Full sample	*n* = 1,162	*n* = 1,203	*n* = 1,138	
Sex (n, %)^a^				
Female	619 (53)	647 (54)	606 (53)	606 (53)
Male	543 (47)	556 (46)	532 (47)	532 (47)
Age (y, mean ± SD)	60.7 ± 9.4	70.0 ± 9.3	60.7 ± 9.4	70.1 ± 9.2
Category of age (n, %)				
45–54	352 (30)	13 (1)	347 (30)	12 (1)
55–64	375 (32)	375 (31)	368 (32)	347 (30)
65–74	330 (28)	411 (34)	320 (28)	396 (35)
75–84	105 (9)	317 (26)	103 (9)	299 (26)
85–94	---	87 (7)	—	84 (7)
Race/ethnicity (n, %)^a^				
African American	330 (28)	337 (28)	319 (28)	319 (28)
Hispanic	509 (44)	526 (44)	503 (44)	503 (44)
White	323 (28)	340 (28)	316 (28)	316 (28)
Education (n, %)^a^				
≤High school degree	482 (42)	486 (40)	467 (41)	467 (41)
Some college	344 (30)	360 (30)	340 (30)	340 (30)
≥College degree	355 (29)	355 (30)	330 (29)	330 (29)
Household income				
$0-$24,999	370 (32)	398 (34)	359 (32)	375 (34)
$25,000-$49,999	387 (34)	334 (29)	379 (34)	319 (29)
$50,000+	391 (34)	442 (38)	386 (34)	416 (37)
GRS	0.51 (0.13)	0.51 (0.13)	—	—

a Variables fixed, reported only at baseline.

**FIGURE 1:**
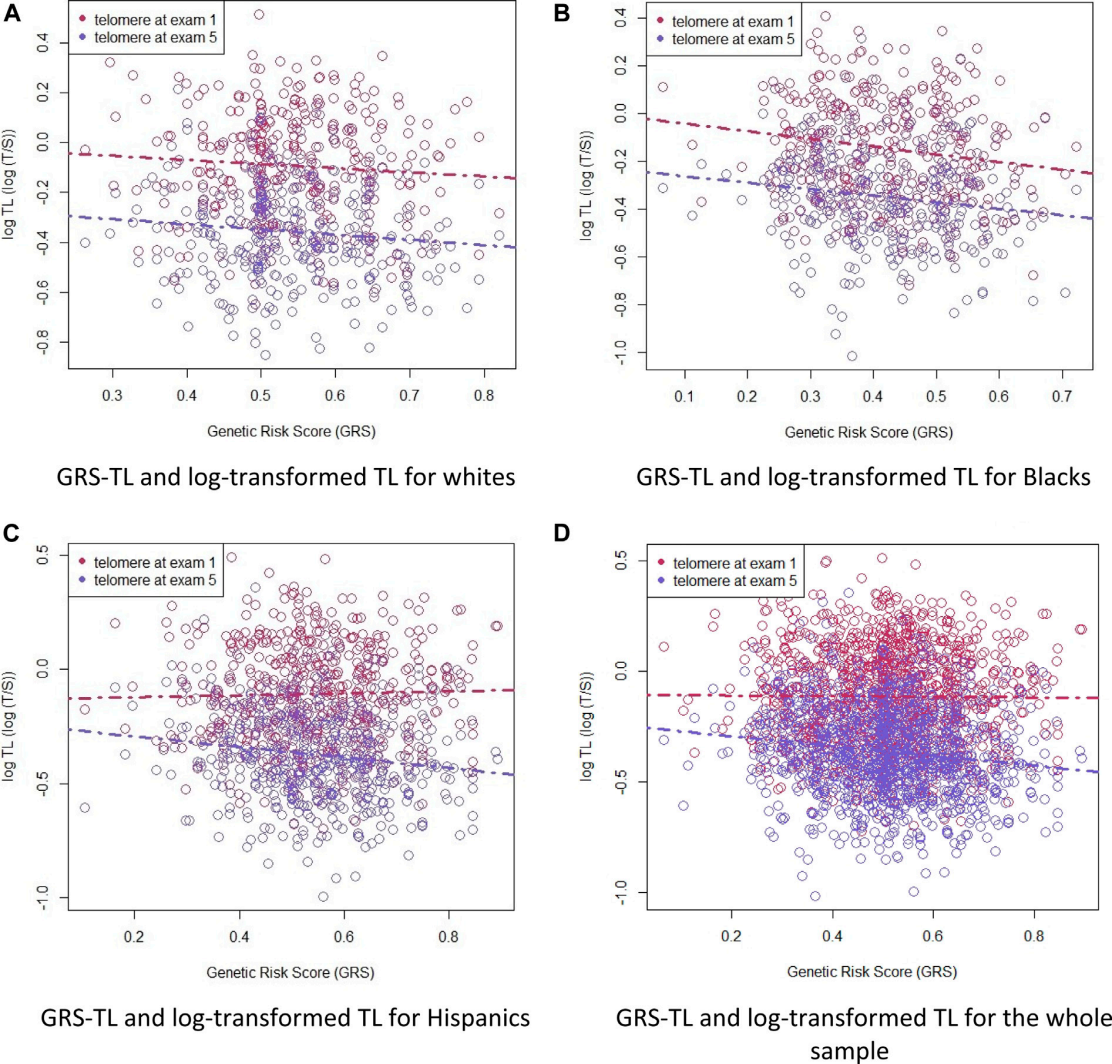
Plots of GRS-TL and TL (T/S ratio) at Exams 1 and 5 by race/ethnicity (white, African American, and Hispanic) and for the whole sample combined. Telomere length as a function of the GRS-TL in Exam 1 (red circles, n = 1162) and Exam 5 (blue circles, n = 1203). Telomere length is plotted as log T/S ratio. Panel (A): GRS-TL and log-transformed TL for whites. Panel (B): GRS-TL and log-transformed TL for Blacks. Panel (C): GRS-TL and log-transformed TL for Hispanics. Panel (D): GRS-TL and log-transformed TL for the whole sample.

We calculated the effect allele frequencies of the SNPs in each race/ethnic group and in all race/ethnicity groups combined and compared them to the effect allele frequencies from the GWAS (Codd et al., 2013), from which we obtained the effect size of the SNPs used to create our GRS-TL (Table 2). The calculated GRS-TL was slightly skewed (Shapiro Wilcoxon *p* = 0.07 and Kolmogorov-Smirnov *p* < 0.01) (Supplementary Figure S1) with a mean (SD) = 0.51 (0.13). Distribution of the GRS-TL for the TA sample and by race/ethnicity is shown in Supplementary Table S1 **.** Hispanics and whites had a greater GRS-TL mean (0.55 and 0.54, respectively) than African American participants (0.42). Plots of GRS-TL and TL (T/S ratio) at Exams 1 and 5 by race/ethnicity and for the whole sample combined are presented in Figure 1.

**TABLE 2:** Effect Allele Frequencies SNPs included in the GRS-TL.

	Effect allele frequency				
SNP	GWAS^a^	White	African american	Hispanic	Full sample
rs10936599	0.25	0.20	0.07	0.30	0.21
rs11125529	0.86	0.89	0.80	0.88	0.86
rs2736100	0.51	0.48	0.53	0.42	0.54
rs755017	0.87	0.90	0.71	0.81	0.81
rs7675998	0.22	0.24	0.20	0.22	0.22
rs8105767	0.71	0.69	0.49	0.62	0.60
rs9420907	0.87	0.84	0.47	0.78	0.71

a Codd et al. (Codd, Nelson et al., 2013).

As expected, we observed a decreasing TL over time. At Exam 1, the average TL (T/S ratio) was 0.92 (SD = 0.21), and at Exam 5 TL was 0.71 (SD = 0.14). Ten-year TA was –0.22 (SD = 0.19). The 10-year TA by demographics is shown in Table 3. Older participants and men had significantly shorter TL at both Exams 1 and 5, but the 10-year TA was not significantly different by age or sex. African Americans had significantly shorter TL than whites at Exam 1, but TL was not significantly different by race/ethnicity at Exam 5; however, 10-year TA was greater for whites and Hispanics than for African Americans. In the whole sample, 83.3% of participants experienced at least a 5% decrease in TL between Exams 1 and 5, whereas only 6.4% experienced at least a 5% increase in TL.

**TABLE 3:** Mean Telomere Length (TL, T/S Ratio) and 10-year Telomere Attrition (10-year TA) for the Full Sample and by demographics.

		TL (exam 1)		TL (exam 5)		10-year TA	
	n	Mean (SD)	p-value	Mean (SD)	p-value	Mean (SD)	p-value
10-Year TA sample	1,138	0.92 (0.21)	—	0.71 (0.14)	—	−0.22 (0.19)	—
By sex	—	—	0,003	—	0.001	—	0.18
Female	606	0.94 (0.20)	—	0.72 (0.15)	—	−0.23 (0.18)	—
Male	532	0.89 (0.21)	—	0.69 (0.14)	—	−0.21 (0.21)	—
By age at Exam 1	—	—	<0.0001	—	<0.0001	—	0.13
45–54	347	0.98 (0.22)	—	0.76 (0.15)	—	−0.23 (0.20)	—
55–64	368	0.94 (0.19)	—	0.71 (0.13)	—	−0.23 (0.19)	—
65–74	320	0.86 (0.19)	—	0.67 (0.14)	—	−0.20 (0.19)	—
75–84	103	0.81 (0.18)	—	0.63 (0.10)	—	−0.20 (0.17)	—
By Race/ethnicity	—	—	0.01^a^	—	0.20	—	<0.001^b^
African American	319	0.89 (0.20)	—	0.72 (0.15)	—	−0.18 (0.19)	—
Hispanic	503	0.92 (0.21)	—	0.70 (0.14)	—	−0.23 (0.19)	—
White	316	0.94 (0.21)	—	0.71 (0.14)	—	−0.24 (0.20)	—
By Education	—	—	0.36	—	0.24	—	0.24
≤High school degree	467	0.92 (0.21)	—	0.70 (0.15)	—	−0.23 (0.20)	—
Some college	340	0.93 (0.21)	—	0.72 (0.14)	—	−0.22 (0.21)	—
≥College degree	330	0.91 (0.19)	—	0.71 (0.14)	—	−0.20 (0.18)	—
Household income	—	—	0.39	—	0.006	—	0.52
$0-$24,999	359	0.91 (0.21)	—	0.69 (0.13)	—	−0.23 (0.19)	—
$25,000-$49,999	379	0.92 (0.21)	—	0.71 (0.15)	—	−0.21 (0.20)	—
$50,000+	386	0.93 (0.20)	—	0.72 (0.15)	—	−0.22 (0.19)	—

10-year telomere attrition = 10*(TL, _exam 5_—TL, _exam 1_)/Visit date _Exam 5_—Visit date _Exam 1_).

a Significant difference between African Americans and whites.

b Difference between African Americans and whites and African Americans and Hispanics.

**FIGURE 2:**
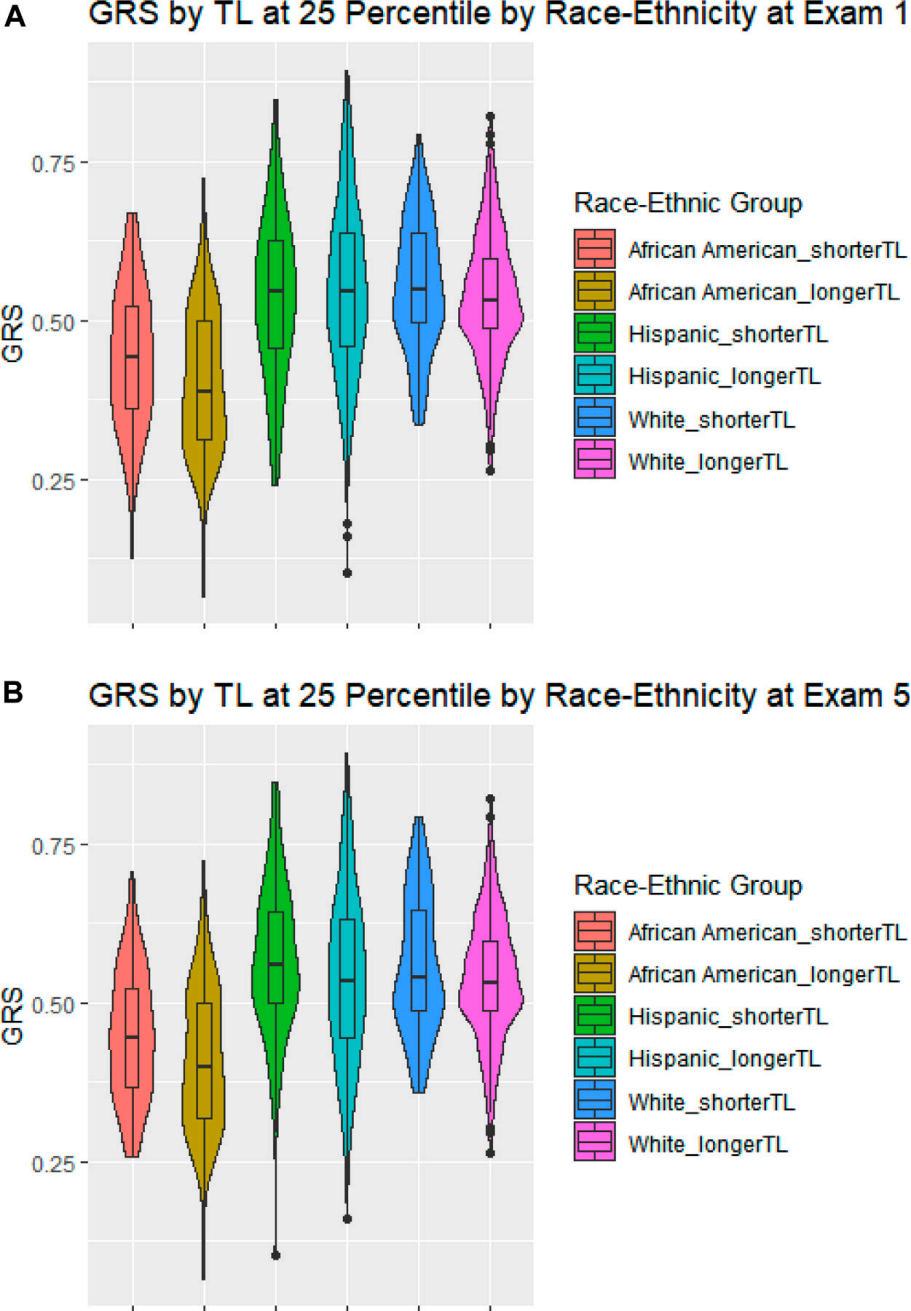
Violin Plots of the GRS-TL distributions stratified by race/ethnicity. Violin Plots of the GRS-TL distributions including a box plot with median, and quartiles stratified by race/ethnicity for those at or below the 25th percentile of TL (shorter TL) and those above the 25th percentile of TL (longer TL). Panel **(A)** GRS by TL at 25 Percentile by Race-Ethnicity at Exam 1; Panel **(B)** GRS by TL at 25 Percentile by Race-Ethnicity at Exam 5.

Figure 2 shows violin plots of the GRS-TL distributions at Exam 1 (Panel A) and Exam 5 (Panel B) stratified by race/ethnicity, comparing those in the lower 25th percentile of TL (shortest) to those in the upper 75th percentile (longest). Although median GRS-TL was higher in those with the lower 25th percentile of TL for most race/ethnic groups at both exams, the GRS-TL distributions between the lower 25th percentile and upper 75th percentile did have substantial overlap.

### TL analysis

In stratified analysis by race/ethnicity, higher GRS-TL was significantly associated with shorter TL among whites, African Americans, and Hispanics. A one-unit increase in the GRS-TL from its mean was associated with a 0.18 unit decrease in T/S ratio (b = –0.18; SE = 0.08; *p* ≤ 0.02 and b = –0.18; SE = 0.07; *p* ≤ 0.01) in whites and African Americans, respectively, and a 0.13 unit decrease in T/S ratio (b = –0.13; SE = 0.05; *p* = 0.02) in Hispanics, after adjustment for exam year, age at exam, sex, and the first three PCs specific for each race/ethnicity (Table 4). In a secondary analysis, we combined all races/ethnicities. The combined sample also showed a significant inverse association between the GRS-TL and TL (estimate = –0.16 [SE = 0.04], *p* < 0.0001) after adjustment for exam year, age, sex, race/ethnicity, and the three PCs of the combined sample. Neither the interaction term between the GRS-TL and African American or GRS-TL and Hispanic were significant (Supplementary Table S2), suggesting that the association between GRS-TL and TL did not differ by race/ethnicity**.**

**TABLE 4:** Association between GRS-TL and TL (T/S ratio) by race/ethnicity.

Race/Ethnicity	Model 1				Model 2		
	Estimate	SE	p-value	Estimate		SE	p-value
White							
Intercept	0.95	0.02	<0.0001	1.01		0.05	<0.0001
Exam 5	−0.22	0.01	<0.0001	−0.22		0.01	<0.0001
Age	−0.05	0.01	<0.0001	−0.05		0.01	<0.0001
Male	−0.04	0.02	0.01	−0.04		0.02	0.01
GRS-TL	−0.19	0.08	0.02	-0.18		0.08	0.02
PC1	0.001	0.02	0.96	0.0004		0.02	0.99
PC2	−0.02	0.07	0.76	−0.02		0.07	0.81
PC3	—	—	—	0.19		0.15	0.19
African American							
Intercept	0.87	0.01	<0.0001	0.86		0.01	<0.0001
Exam 5	−0.17	0.01	<0.0001	−0.17		0.01	<0.0001
Age	−0.06	0.01	<0.0001	−0.06		0.01	<0.0001
Male	−0.04	0.01	0.001	−0.04		0.01	0.003
GRS-TL	−0.19	0.07	0.004	−0.18		0.07	0.01
PC1	−0.02	0.05	0.74	−0.02		0.05	0.73
PC2	−0.08	0.05	0.13	−0.09		0.05	0.10
PC3	—	—	—	0.10		0.06	0.09
Hispanic							
Intercept	0.95	0.01	<0.0001	0.96		0.01	<0.0001
Exam 5	−0.22	0.01	<0.0001	−0.22		0.01	<0.0001
Age	−0.05	0.01	<0.0001	−0.05		0.01	<0.0001
Male	−0.05	0.01	<0.0001	−0.05		0.01	<0.0001
GRS-TL	−0.13	0.05	0.01	−0.13		0.05	0.02
PC1	−0.18	0.04	<0.0001	−0.18		0.04	<0.0001
PC2	0.12	0.03	0.0002	0.12		0.03	0.0002
PC3	—	—	—	−0.05		0.04	0.26

SE, standard error; TL, telomere length (T/S ratio).

GRS-TL, age, gender, and PCs, were centered to their population mean.

Exam 5 (referent = Exam 1); male (referent = female); principal components: PC1, PC2 and PC3 specific for each race/ethnicity.

Model 1 control for study exam (Exam 1 vs. 5), age at exam, and gender plus PC1 and PC2.

Model 2 include PC3 in addition of variables in model 1.

### TA analysis

In stratified analysis by race/ethnicity, GRS-TL was not significantly associated with 10-year TA for any race/ethnic group (Table 5). Results were similar in the combined sample (Supplementary Table S3).

**TABLE 5:** Analysis of the association of GRS-TL with 10-year Telomere Attrition (10-year TA) stratified by race/ethnicity.

Race/Ethnicity	Model 1				Model 2		
	Estimate	SE	p-value	Estimate		SE	p-value
White							
Intercept	0.82	0.01	<0.0001	0.82		0.01	<0.0001
Time	−0.30	0.04	<0.0001	−0.30		0.04	<0.0001
Time x GRS-TL	0.06	0.12	0.60	0.06		0.12	0.58
Time x age	0.01	0.01	0.28	0.01		0.01	0.27
Time x male	−0.02	0.02	0.41	−0.02		0.02	0.42
Time x PC1	0.00	0.03	0.92	0.00		0.03	0.91
Time x PC2	0.12	0.08	0.15	0.12		0.08	0.15
Time x PC3	—	—	—	0.04		0.14	0.76
African American							
Intercept	0.81	0.01	<0.0001	0.81		0.01	<0.0001
Time	−0.20	0.03	<0.0001	−0.21		0.03	<0.0001
Time x GRS-TL	0.14	0.09	0.14	0.14		0.09	0.13
Time x age	0.02	0.01	0.05	0.02		0.01	0.05
Time x male	0.04	0.02	0.10	0.04		0.02	0.09
Time x PC1	−0.08	0.07	0.23	−0.08		0.07	0.23
Time x PC2	0.08	0.08	0.28	0.08		0.08	0.29
Time x PC3	—	—	—	0.04		0.08	0.64
Hispanic							
Intercept	0.81	0.01	<0.0001	0.81		0.01	<0.0001
Time	−0.30	0.02	<0.0001	−0.28		0.03	<0.0001
Time x GRS-TL	−0.04	0.07	0.60	−0.03		0.07	0.61
Time x age	0.01	0.01	0.26	0.01		0.01	0.26
Time x male	0.03	0.02	0.05	0.03		0.02	0.05
Time x PC1	0.28	0.05	<0.0001	0.28		0.05	<0.0001
Time x PC2	−0.001	0.04	0.99	−0.002		0.04	0.97
Time x PC3	—	—	—	−0.06		0.06	0.37

Time (follow-up time) was centered to the individual’s mean follow-up time; all other variables were centered at population mean.

Age, age at exam 1; GRS-TL, genetic risk score for TL; PC, principal components specific for each race/ethnicity.

A negative coefficient for an interaction with time indicates greater 10-year telomere attrition.

Model 1 controls for follow-up time, age, and gender in interaction with follow-up time, PC1 and PC2, and model 2 further includes PC3.

## Discussion

The goals of the current study were 1) to examine the association between genetic predisposition to shorter TL, measured by a weighted GRS of seven SNPs, and TL and 2) to examine the association between the GRS-TL and within-person TA over 10 years. In our study, we observed an association between the GRS-TL and TL in each of the race/ethnic groups (white, African American, and Hispanic). When we analyzed the whole sample (all three race/ethnicity groups combined), the association between the GRS-TL and TL was also significant in the fully adjusted model. However, the GRS-TL was not associated with the risk of 10-year TA.

The heritability (h^2^) estimate of TL in a meta-analysis of six cohorts, all of European descent, was 0.70 (95% confidence interval 0.64–0.76). (Broer et al., 2013). Other cohorts have showed greater variation of h^2^ of TL. (Dugdale and Richardson, 2018). The h^2^ of TA has been less investigated, and it seems lower than the h^2^ of TL. In a study using same-sex twin data, h^2^ of TA was between 24% and 32% with low or no shared environmental effect. (Hjelmborg et al., 2015). The authors of this study suggested that heritability and early life environment may determine the rate of TA early in life, before adulthood, and this could be related to why TA is much faster in children than in adults. (Hjelmborg et al., 2015).

### GRS for TL

In the current study, we observed a significant inverse association between a weighted GRS of seven SNPs for shorter TL, which was derived from a GWAS in samples of European descent, with TL in African Americans, Hispanics, and whites. In the combined sample, there was also a significant inverse association between the GRS-TL and TL in the fully adjusted model, and the association between GRS-TL and TL did not differ by race/ethnicity.

Replication studies in different race/ethnic backgrounds have showed mixed results. A GWAS meta-analysis in mostly white and a small group of African Americans participants identified associations of TL with two loci: OBFC1(rs4387287, rs9419958, and rs2487999) and CXCR4 (rs4452212 and 4954585). Further, evidence of replication was found for rs4387287 and rs9419958 in whites and a combined sample of whites and African Americans, but not in African Americans alone. The other SNPs were not replicated in any group (whites, African Americans, or both races combined). (Levy et al., 2010). Another study sought replication in only two out of their seven SNPs associated with short TL in a pooled cohort of participants of European descent. (Codd et al., 2013).

In a sample of 11,934 older adults from the US Health and Retirement Study (76% white, and 24% African Americans, Hispanics, or other race/ethnicity), a GRS-TL derived from the same GWAS we used in the current study was evaluated. In that study, compared to whites, African Americans were significantly more likely to have a lower GRS-TL, but Hispanics were more likely to have a higher GRS-TL. However, after adjustment for population structure, these associations were no longer significant. (Hamad et al., 2016). Hamad et al. also found differences in the association of individual TL-associated SNPs and TL by race/ethnicity for some SNPs. (Hamad et al., 2016).

### Strengths and limitations

The current study has several strengths. First, this is a large community-based study that includes three race/ethnic groups (whites, African Americans, and Hispanics), with an overrepresentation of the last group as opposed to what is observed in other studies, whose percent of Hispanics were small. (Hamad et al., 2016). Second, the average inter-assay coefficient of variation for the telomere length assay was only 2.9%. Third, our GRS was estimated on qPCR, which is a better choice for our current study, but future work will examine sequencing-estimated scores. (Cawthon, 2002; Codd et al., 2013). Fourth, our analytical approach included LMEM, which allowed us to use our data more efficiently. Finally, the period between the first and second telomere length measurements was 10 years, which is about the time needed between measurements to observe true change in TL in adults, as reported in previous research. (Steenstrup et al., 2013). Whereas most participants in our sample experienced a decrease in TL between Exams 1 and 5, 6.4% experienced an increase in TL of at least 5%. Although we cannot rule out the possibility that measurement error accounts for this finding, a simulation study by Bateson and Nettle (Bateson and Nettle, 2017) indicates that scenarios in which true lengthening occurs provide a better fit for the empirical data than scenarios in which no lengthening occurs. In addition, prior experimental studies have shown that non-technical factors, such as weight loss (García-Calzón et al., 2014; Carulli et al., 2016) may contribute to telomere lengthening.

Our study also has several limitations. First, to create our GRS-TL, we used 7 SNPs derived from a meta-analysis GWAS in samples of European descent, which almost certainly has greater predictive utility for whites than for African Americans or Hispanics. Second, the genetic factors we used to create our GRS-TL may influence only telomere length, but the rate of TA may be influenced by different genetic factors, as mentioned by others. (Levy et al., 2010). Sanders et al. suggest that TL indicates somatic growth before the end of puberty and cellular senescence and oxidative stress after puberty. (Sanders and Newman, 2013). TL may be affected by DNA lost during replication and by oxidative stress, (Proctor and Kirkwood, 2002), but also by several environmental factors such as smoking and low socioeconomic status. (Needham et al., 2013). The genetic and environment correlates of TL may differ from the genetic and environment correlates of TA. Third, TA is difficult to study, particularly when using PCR, because it is measured with error, which could explain why the GRS-TL was not significant in the TA analysis. (Nettle et al., 2021). Fourth, the period between the first and second measurement may not be long enough to observe a meaningful change in TL given that the average annual rate of TL shortening in adults is slower than it is before the 20 years of age. Last, the range of age of our sample did not include very young participants where we could have observed greater TA.

## Conclusion

In conclusion, our study focused on the ability to evaluate whether SNPs identified in the European GWAS were associated with TL and 10-year TA in a multi-ethnic sample. In the TL analysis, we observed an association between GRS-TL and TL in each of the race/ethnic groups studied, with a similar degree of association in whites and African Americans and a little lower among Hispanics. The association was also present and significant when we combined the race/ethnic groups, suggesting that the GRS-TL is associated with TL well in the three race/ethnic groups. However, we did not find an association between our GRS-TL and 10-year TA and this could be explained because the SNPs used in our GRS-TL reflect variants of telomere length but not of telomere shortening over some time. Larger longitudinal studies that include a wide range of age from birth to old age may be needed to better understand the dynamics of telomere length.

## Data Availability

The data analyzed in this study is subject to the following licenses/restrictions: “The MESA data used to support the findings of this study are available at the Multi-ethnic Study of Atherosclerosis website https://www.mesa-nhlbi.org at request after following procedures”. Requests to access these datasets should be directed to Belinda Needham, needhamb@umich.edu.
