# Supplementary material for: Prediction of telomere length and telomere attrition using a genetic risk score: The multi-ethnic study of atherosclerosis (MESA)
Source: Front Aging. 2022 Oct 11;3:1021051. doi: 10.3389/fragi.2022.1021051 (PMC9592760; doi:10.3389/fragi.2022.1021051)
Supplement: Supplementary file 1 [file DataSheet1.docx]

**Supplementary Materials:**

**Table S1.** Distribution of the Genetic Risk Score for TL (GRS-TL)

**Table S2**. Association between GRS-TL and TL (T/S ratio) all race/ethnicities combined

**Table S3**. Analysis of the association of GRS-TL with 10-year Telomere Attrition (10-year TA) all race/ethnicities combined

**Table S1.** Distribution of the Genetic Risk Score for TL (GRS-TL)

|  | n | Mean | Standard deviation | Median | Lower Quartile | Upper Quartile | Minimum | Maximum |
| --- | --- | --- | --- | --- | --- | --- | --- | --- |
| Full sample | 1138 | 0.51 | 0.13 | 0.51 | 0.42 | 0.59 | 0.07 | 0.89 |
| Race/Ethnicity |  |  |  |  |  |  |  |  |
| White | 316 | 0.54 | 0.10 | 0.54 | 0.49 | 0.60 | 0.26 | 0.82 |
| African American | 319 | 0.42 | 0.11 | 0.41 | 0.33 | 0.51 | 0.07 | 0.72 |
| Hispanic | 503 | 0.55 | 0.13 | 0.55 | 0.46 | 0.63 | 0.10 | 0.89 |

**Table S2**. Association between GRS-TL and TL (T/S ratio) all race/ethnicities combined

|  | TL | | |  |
| --- | --- | --- | --- | --- |
|  | Estimate | SE | p-value | |
| Intercept | 0.92 | 0.01 | **<.0001** | |
| Exam 5 | -0.21 | 0.01 | **<.0001** | |
| Age at Exam 1 | -0.05 | 0.004 | **<.0001** | |
| Male | -0.04 | 0.01 | **<.0001** | |
| African Americans | -0.07 | 0.02 | **0.003** | |
| Hispanics | -0.08 | 0.02 | **<.0001** | |
| GRS-TL | -0.16 | 0.04 | **<.0001** | |
| African Americans x GRS-TL | -0.004 | 0.10 | 0.97 | |
| Hispanics x GRS-TL | 0.06 | 0.09 | 0.53 | |
| PC1 | -0.002 | 0.03 | 0.95 | |
| PC2 | 0.062 | 0.104 | 0.55 | |
| PC3 | 0.19 | 0.104 | 0.07 | |

SE= standard error TL=telomere length (T/S ratio)

Exam 5 (referent = Exam 1); male (referent = female); race-ethnicity (African Americans, Hispanics, and whites (referent group)) principal components (PC)s: for the whole sample

Gender, age, race/ethnicity, GRS-TL, and principal components (PC)s were all centered to the population mean

Model controls for study exam (Exam 1 vs. 5), age at exam, gender, and race/ethnicity plus interaction terms race-ethnicity x GRS-TL plus PC1, PC2 and PC3.

**Table S3**. Analysis of the association of GRS-TL with 10-year Telomere Attrition (10-year TA) all race/ethnicities combined

|  | 10-year TA | | | |
| --- | --- | --- | --- | --- |
|  | Estimate | SE | p-value |  |
| Intercept | 0.81 | 0.004 | **<.0001** |  |
| Time | -0.20 | 0.02 | **<.0001** |  |
| Time x GRS-TL | 0.03 | 0.05 | 0.52 |  |
| Time x age | 0.01 | 0.01 | **0.02** |  |
| Time x male | 0.02 | 0.01 | 0.09 |  |
| Time x African Americans | -0.01 | 0.03 | 0.85 |  |
| Time x Hispanics | 0.04 | 0.02 | 0.09 |  |
| Time x GRS-TL x African Americans | 0.10 | 0.15 | 0.49 |  |
| Time x GRS-TL x Hispanics | -0.09 | 0.13 | 0.49 |  |
| Time x GRS-TL x PC1 | 0.13 | 0.04 | **.001** |  |
| Time x GRS-TL x PC2 | -0.02 | 0.07 | 0.81 |  |
| Time x GRS-TL x PC3 | -0.18 | 0.08 | 0.03 |  |

Time (follow-up time) was centered to the individual’s mean follow-up time; all other variables were centered at population mean

Age = age at exam 1; GRS-TL = genetic risk score for TL; PC= principal components (PC1 and PC2) for the combined sample

A negative coefficient for an interaction with time indicates greater 10-year telomere attrition.

Model controls for follow-up time, age, gender, and race/ethnicity, 3-way interaction terms: follow-up time, GRS-TL and race/ethnicity plus 3-way interaction terms: follow-up time, GRS-TL, and PCs
