# Supplementary figures and images for: Prediction of telomere length and telomere attrition using a genetic risk score: The multi-ethnic study of atherosclerosis (MESA)
Source: Front Aging. 2022 Oct 11;3:1021051. doi: 10.3389/fragi.2022.1021051 (PMC9592760; doi:10.3389/fragi.2022.1021051)

**Figure S1.** Histogram of the Genetic Risk Score for Telomere Length

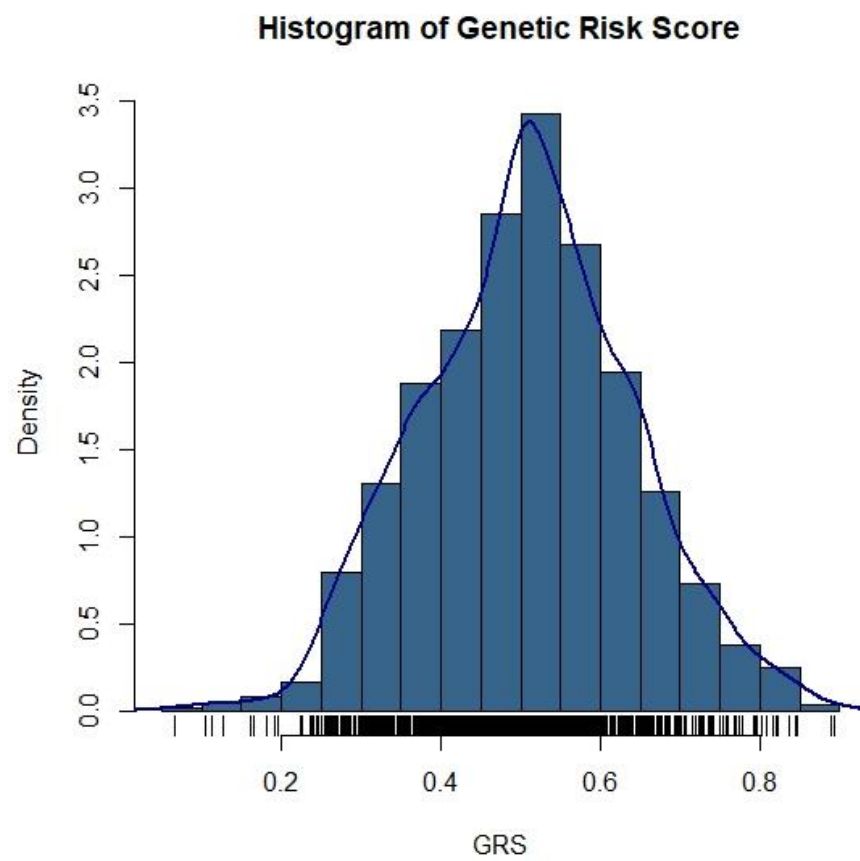

Supplement: Supplementary file 2 [file Image1.pdf]
